# Virtual Sensors for On-line Wheel Wear and Part Roughness Measurement in the Grinding Process

**DOI:** 10.3390/s140508756

**Published:** 2014-05-19

**Authors:** Ander Arriandiaga, Eva Portillo, Jose A. Sánchez, Itziar Cabanes, Iñigo Pombo

**Affiliations:** 1 Department of Automatic Control and System Engineering, University of the Basque Country, C/Alameda Urquijo s/n, 48013 Bilbao, Spain; E-Mails: eva.portillo@ehu.es (E.P.); itziar.cabanes@ehu.es (I.C.); 2 Department of Mechanical Engineering, University of the Basque Country, C/Alameda Urquijo s/n, 48013 Bilbao, Spain; E-Mails: joseantonio.sanchez@ehu.es (J.A.S.); inigo.pombo@ehu.es (I.P.)

**Keywords:** virtual sensor, grinding process, wheel wear, surface roughness, Artificial Neural Networks

## Abstract

Grinding is an advanced machining process for the manufacturing of valuable complex and accurate parts for high added value sectors such as aerospace, wind generation, *etc*. Due to the extremely severe conditions inside grinding machines, critical process variables such as part surface finish or grinding wheel wear cannot be easily and cheaply measured on-line. In this paper a virtual sensor for on-line monitoring of those variables is presented. The sensor is based on the modelling ability of Artificial Neural Networks (ANNs) for stochastic and non-linear processes such as grinding; the selected architecture is the *Layer*-*Recurrent* neural network. The sensor makes use of the relation between the variables to be measured and power consumption in the wheel spindle, which can be easily measured. A sensor calibration methodology is presented, and the levels of error that can be expected are discussed. Validation of the new sensor is carried out by comparing the sensor's results with actual measurements carried out in an industrial grinding machine. Results show excellent estimation performance for both wheel wear and surface roughness. In the case of wheel wear, the absolute error is within the range of microns (average value 32 μm). In the case of surface finish, the absolute error is well below *R_a_* 1 μm (average value 0.32 μm). The present approach can be easily generalized to other grinding operations.

## 1. Introduction

Abrasive material removal processes are nowadays key technologies in modern manufacturing. Amongst them, grinding has become critical despite of the advances in the performance and accuracy of other manufacturing processes, such as turning or milling. It is due to its capacity for producing parts of high precision and high surface quality in difficult-to-machine materials that grinding is widely used in the motor and aerospace industries and by precision cutting tool manufacturers [1], amongst others. The application in those high-added value sectors easily explains why grinding has been the object of extensive research during the past 40 years and it is still nowadays.

Ground components are characterized by their extremely tight dimensional tolerances and very low surface finish, in many cases well below *R_a_* 0.2 μm. Grinding is, therefore, a high-accuracy technology in which process set-up, control and monitoring are of primary importance if strict customer requirements are to be met economically. It must be taken into account that in the past decade, about 20%–25% of the total cost of all machining processes was due to grinding [1].

Setting-up of the grinding process involves time and cost-consuming operations. Theoretical models have been developed, but industrial application is not an easy task yet. Theoretical models require calibration before they can be used in practical grinding operations. Examples of such models can be found in [2]. Commercial software has been developed under this approach (for instance, the Gindsim^©^ software developed by Malkin [2]).

Control and monitoring of the results of the grinding process is severely limited by the low accessibility of the machining area. The high rotational speed of the grinding wheel (common values are around 45 m/s, but with industrial examples of as much as 200 m/s), the generation of abrasive swarf, the presence of large quantities of coolant at high pressures, *etc.* all combine to limit the possibility of using industrial sensors during the process. Commercial industrial sensors can be found in the field of the measurement of workpiece dimensional tolerances and in the use of Accoustic Emission (AE) for contact detection [3], for instance. Also, attempts have been made to quantify wheel wear during grinding process execution [4,5], but still, the evolution with time of critical process variables such as grinding wheel wear, workpiece surface finish and workpiece burn (amongst others) remain as current research topics [6] or even, in some of those cases, remain unaddressed.

The limitations of theoretical explicit models for the prediction of grinding wheel wear and of the final workpiece surface finish can be understood at the view of the following reasons: the important lack of precise information about the composition of the wheel itself and its performance must be mentioned first. Then, the relations between the different process variables and process outputs are highly non-linear. Finally, the accuracy required in the final results (on the order of tenths of microns in the case of surface roughness) must also be taken into account. All these facts have led many researchers to use Artificial Intelligence techniques in order to optimize process modelling with respect to the results provided by explicit theoretical models.

Using intelligent techniques for estimation and/or prediction of outputs of the grinding process is a well-known approach. In most of the cases the output parameter is surface roughness, although there are a number of research works in which wheel wear was studied.

A Fuzzy Logic controller was designed in [7] for the prediction of the final surface finish and the required spindle power in the grinding of steel using a grinding wheel as given by the standard specification D126K5V. The controller was optimized in a later research work by applying Genetic Algorithm (GA) techniques. In [8], prediction of surface finish in creep-feed grinding using an aluminum oxide wheel with grit size 60 was carried out through a multi-layer perceptron Neural Network. Application of Artificial Neural Networks (ANNs) was also presented in [9] for prediction of surface finish in creep-feed grinding. In this case, once surface finish is known, GA techniques are used for maximization of material removal rate and minimization of wheel wear. Genetic Algorithm techniques have also been used for training an ANN aiming at predicting the maximum surface roughness (peak to valley value) in surface grinding [10]. In a more recent work, a similar approach has been presented by other authors [11] for the prediction of surface finish in cylindrical grinding of steel parts using a GB70RAP400 grinding wheel. In [12], Maksoud *et al.* describe the methodology for implementation of a grinding process controller for surface grinding based on neural networks. The architecture of the network is explained and application examples about control and redesign of grinding operations are presented. In this work the authors feed the network with wheel characteristics and process variables, as well as with the following data taken in-process: wheel surface characteristics, vertical component of the cutting force and grinding wheel eccentricity. The output of the system is an on-line measure of workpiece surface grinding and the possibility of deciding the redesign of the grinding process. Still, the approach requires the use of expensive sensors such as the dynamometric table, which can be used in laboratory tests but cannot be extensively implemented in an industrial environment. Also, the use of the HRPS sensor requires modification of the wheel spindle. In a later work [13], the same authors concluded that much of the prior research used artificial intelligence algorithms for process design systems, but the authors stated that the developed systems do not respond dynamically to the values measured on-line. Therefore, research is still required to advance in this field.

When it comes to the study of wheel wear, nearly all research works are devoted to the use of Intelligent Techniques to distinguish between a sharp or dull wheel. In most of the cases, information is taken from AE sensors. In [14] a neuro-fuzzy system for cylindrical grinding is described. The ANN was used for reducing the number of inputs to the system, and the fuzzy system was then applied to decide whether the wheel was in good or in poor condition. Validation is shown for a 38A80KVBE grinding wheel. In [15] the feasibility of the Hidden Model of Markov (HMM) to predict the state of SD220R75B56-1/8 and SD220R100B99-1/8 diamond grinding wheels during creep-feed grinding was presented. In a later work [16], the research was completed by using automatic boosting training in the ANN. In both works, the AE signal was pre-processed using the wavelet transformation. Support Vector Machines (SVM) have also been proposed as s classification method [17].

A literature review reveals that ANNs constitute, in fact, an effective approach to grinding process modelling. However, all the research works found in scientific literature provide particular solutions for a given wheel-workpiece pair. Results cannot be, in any case, generalized to other types of wheels. In fact, in most of the cases, they cannot even be generalized to grinding conditions that have not been used during the training of the network. This is one of the reasons why industrial application of these techniques in grinding has been very limited so far. In the cases of prediction of surface finish, the output of the network is the value related to the current state of wear of the grinding wheel. However, it is well-known in industrial practice that the surface roughness of the ground components changes over time as the wheel loses its cutting ability.

A step forward can be found in the use of ANNs as virtual sensors. Although during the last years the design and development of hardware-based sensors has shown impressive improvements, more and more ANNs are being used as soft sensors in different application fields due to their capacity to solve non-linear problems [18] and to process information of different nature [19].

The literature review shows that on-line monitoring of the grinding process is a topic of interest both for industry and academy. Solutions for on-line estimation of wheel wear and workpiece surface finish have been presented in the last few years. The development of hard sensors has been complemented with the use of Artificial Intelligence techniques. This work acknowledges the previous research, and tries to go a step forward in the application of Artificial Neural Networks as virtual sensors for the grinding process. In this approach, a very simple and low-cost power meter device (that can be easily implemented in any kind of grinding machine) is used as on-line input signal for the virtual sensor, avoiding thus the need for expensive force measuring devices (such as the dynamometric table) or the need for machine mechanical modification. Using the well-known equations presented in Section 2, the measurement of power consumption can be used for on-line estimation of surface finish and wheel wear.

## 2. Design of an ANN-Based Virtual Sensor for the Grinding Process

This section deals with the conceptual design of the proposed ANN-based virtual sensor from the theoretical knowledge of the grinding process. Then, criteria applied for the selection of the most appropriate ANN architecture are presented. Finally, a methodology for the calibration of the ANN-based sensor is proposed.

### 2.1. Conceptual Design of the Virtual Sensor

As explained above, the starting point for the development of our approach is based on two facts: on the one hand, the need for reliable industrial sensors for the grinding process; on the other, the existence of theoretical models for the prediction of surface roughness and wheel wear in the grinding process. Marinescu [2] collected a number of different expressions for the different outputs of the grinding process. Amongst them, Equations (1) and (2) allow relating specific grinding energy (*e_c_*), surface roughness (*R*) and grinding wheel wear (*V_s_*):

(1) $$e_{c}\approx k\cdot \sqrt{\frac{V_{s}}{V_{w}}\cdot C\cdot r\cdot \sqrt{\frac{d_{e}}{a_{e}}}}$$

(2) $$R\approx {\left(\frac{V_{s}}{V_{w}}\cdot \frac{1}{C\cdot r\cdot \sqrt{d_{e}}}\right)}^{⅔}$$

Equations (1) and (2) show the non-linear dependency of both specific grinding energy and surface roughness with process parameters (speed ratio, depth of cut, wheel diameter) and with the state of wear of the grinding wheel, expressed through the product *C*·*r*, where *C* is grain density and *r* is the so-called grit shape factor. Commonly, *C*·*r* can be considered as a single factor related to the surface topography and surface wear of the grinding wheel. In industrial application there is not any sensor available for the measurement of that product, which makes direct solving of the above expressions impossible in practice.

As far as concerns *e_c_* and *R*, the situation is quite different. The specific grinding energy *e_c_* can be easily and directly measured on-machine from power consumption measurements obtained from the wheel spindle:

(3) $$e_{c}=\frac{P}{Q_{w}}$$

where *P* is the power consumption at the wheel spindle and *Q_w_* is the material removal rate of the operation. On the other hand, the surface roughness *R* of the machined workpieces can only be measured once they have been machined (in other words, measurement of surface finish cannot be accomplished on-machine).

Therefore, a robust and low-cost power meter can be used for on-line measurement of the values of *e_c_*. A novel approach is presented at this point, based on the implementation of a virtual sensor able to obtain the evolution of wheel wear and surface roughness, as the production progresses and the number of machined parts increases, from reliable *e_c_* values measured during grinding. As explained above, during grinding the use of industrial sensors is severely limited due to the very aggressive conditions and low accessibility to the machining area. This is why the design of a new virtual sensor is proposed at this point.

### 2.2. Selection of the Architecture for the Artificial Neural Network

It has been therefore decided to create soft sensors for wheel wear and workpiece surface finish monitoring in grinding using ANN techniques. One of the most important decisions for the proper design of an ANN is to select an appropriate architecture. As presented in Section 1, the prediction of the wheel wear and the surface finish has been accomplished from a static point of view by employing static neural architectures when the neural network approach is adopted. In this type of networks the current outputs depend on the current inputs, *i.e.*, the past values of the grinding variables are not considered. However, in this work the aim is to monitor the evolution of surface finish and wheel wear along time. Consequently, a recurrent neural architecture is to be selected due to the dynamic nature of the problem. In this kind of artificial neural networks past values are fed back to the inputs so as to provide the ANN with memory. Among the most extended recurrent architectures, two types can be distinguished depending on the required real samples during the ANN operation: recurrent neural networks that require initial real values (for instance, Nonlinear AutoRegressive network with eXogenous inputs *NARX*), and recurrent neural networks that do not require initial real values (for instance, *Elman*). Since measuring real values of surface finish and wheel wear is very time-consuming, these measurements are to be avoided. Thus, a recurrent neural architecture that does not require initial real values should be employed. In particular, an Elman-inspired (Equations (4) and (5)) recurrent neural network, the Layer-Recurrent neural network [20] is selected. In both types of architectures the recurrent connections are settled from the output of the hidden layer to the input layer. However, the *Layer*-*Recurrent* architecture provides more flexibility since it makes possible to have more than one hidden layer and different transfer functions in each layer:

(4) $$\text{O}(\text{t})=\text{purelin}({\text{LW}}^{2,1}{\text{a}}_{1}(\text{t})+{\text{b}}_{2})$$

(5) $${\text{a}}_{1}(\text{t})=\text{tansig}({\text{IW}}^{1,1}\text{p}(\text{t})+{\text{LW}}^{1,1}{\text{a}}_{1}(\text{k}-1)+{\text{b}}_{1})$$

where O(t) is the output of the net, a_1_(t) is the output of the second network, W are the weights of each layer and, finally, p(t) is the input to the net.

**Figure 1.**
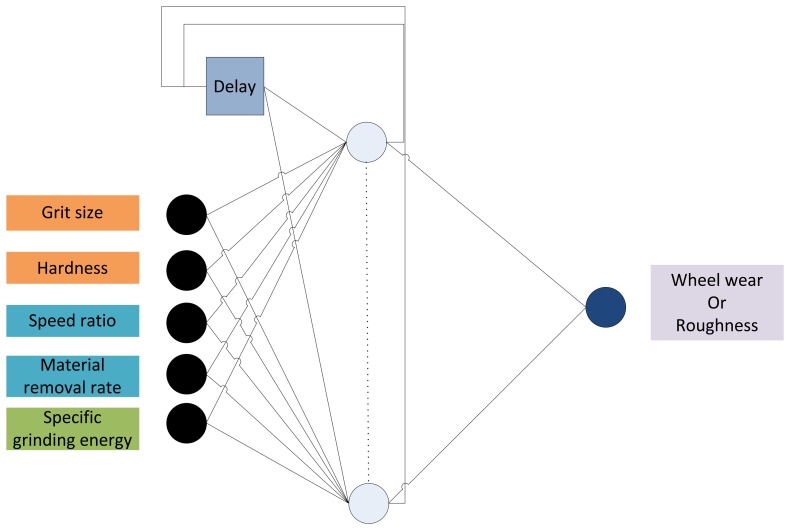
Layer-Recurrent Neural Network for wheel wear and roughness prediction. The input/output *signals* of the sensors are also included.

Considering all these aspects, Figure 1 shows the selected neural *architecture*. It is important to highlight the input *signals* for the soft sensors:

- Characteristics related to grinding wheel specification: grit size and wheel hardness. Both inputs are correlated with the product *C*·*r* (Equations (1) and (2)).
- Grinding process variables: specific material removal rate *Q'* and speed ratio *q_s_*. These inputs are previously adjusted in the machine Computer Numerical Control (CNC).
- Specific grinding energy *e_c_* (see Equations (1) and (3)), which is on-line measured using the power meter installed on the grinding machine.

Since the objective is to monitor surface roughness and wheel wear during a significant period of time, dressing parameters have not been included in the design. It is well-known that the influence of dressing conditions is only evident during the first workpieces ground after dressing, and becomes negligible for a sufficient number of parts machined over time. The inputs for the sensor have been selected by taking into account their relative importance on the grinding process, making use of previous expertise of the research group and of wheel manufacturers and users.

It must be noticed that this proposal is far from the classic use of recurrent ANN in which the input (*y(t*−*1)*, *y(t*−*2)*,…) is given by the past values of the output to be forecasted (*y(t)*, *y(t*+*1)*,…) [21]. In fact, the neural configurations proposed in this work aims at providing time-dependent models, *i.e.*, estimations of the output (*y(t)*) from a different dynamic input (*e(t*−*1)*, *e(t*−*2)*,…), as well as from static cutting parameters adjusted in the CNC and characteristics dependant on the wheel [21]. More detailed information about the inputs is given in Section 3.

### 2.3. Methodology for Sensor Calibration: Training of the ANN

Like any other hardware sensor, the virtual sensor presented in this work requires a proper calibration so as to guarantee the reliability of the measurements. During the calibration of the virtual sensor the *configuration* of the ANN is established, whereas ANN *configuration* is defined by an ANN with a specific number of hidden units *HU* and delays *D*. This aims at defining the dimension and the dynamic behaviour of the ANN.

**Figure 2.**
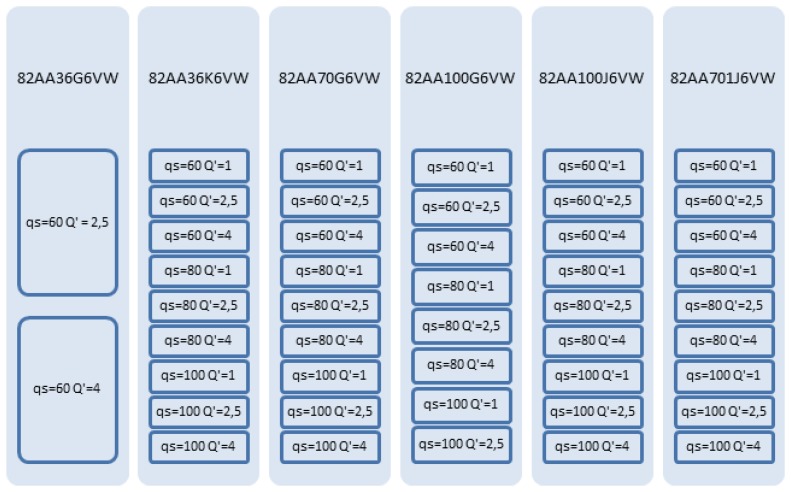
Grinding wheels and grinding conditions of the experimental database for training and testing the ANN-based sensors.

Training an ANN *configuration* involves performing grinding experiments, carried out on an industrial Danobat FG-600-S cylindrical grinding machine. Grinding wheels with different specifications in terms of abrasive grit size and hardness have been selected. The field of application of the sensor must be defined, since grinding operations cover an extremely large number of components and requirements. Since the objective of this work is to show the potential of the methodology, an average area of application related to the grinding of steel parts with non-extremely demanding surface finish has been selected. Figure 2 shows the wheels and grinding conditions used for the training and testing process. A total of 46 experiments have been performed. In all the cases the same alumina grade and wheel structure has been used. If, for instance, the sensor is to be applied in a very demanding grinding operation in terms of surface finish, wheel structure will have to be included as one of the inputs described in Figure 1. However, the methodology for ANN training (and therefore, sensor calibration) would still be exactly the same as the one described here.

Each experiment involves grinding a total amount of workpiece material of 40,000 mm^3^. Usually, grinding variables are referred to the unit wheel width [22]. In this case, since wheel width is 20 mm, the total specific volume of part material removed *V′_w_* in each experiment is, therefore, 2000 mm^3^/mm. For an industrial example, in which competition motorcycle components are ground to the final shape, this value of *V′_w_* corresponds to an approximate number of 1300 machined parts. During each experiment values of surface finish and wheel wear are periodically measured, at different values of *V′_w_*. Wheel wear is known by periodically machining an aluminium plate and comparing the difference in wheel diameter. This is a common technique at lab scale [22]. Likewise, the actual roughness of the machined part is known by using a profilometer. The measured values of both wheel wear and roughness are manually stored in software files known as *experiment files* so as they can be employed in the further training process.

**Figure 3.**
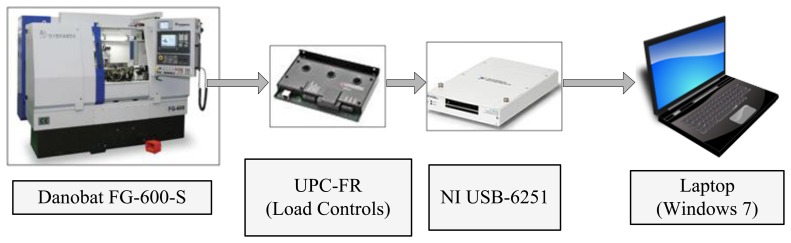
Acquisition system for collecting power samples.

Power consumption *P* (see Equation (3)) at the wheel spindle is measured through a Hall-effect based transducer (UPC-FR by Load Controls). Samples are continuously stored during grinding by a NI USB-6251 board at a sample and storage rate of 100 Hz (see Figure 3).

In order to obtain periodic time-series, in each experiment interpolation techniques have been applied to the sequence of measurements of surface finish and wheel wear. In particular, the *smoothing spline* with a *smoothing parameter* value equal to 1.91 is applied so as to avoid excessive oscillations. The criterion is to yield one value every *V′_w_* equal to 10 mm^3^/mm.

During the training process, the preselected *Layer-Recurrent* architecture is trained with different values of hidden units *HU* and delays *D*, and one unique hidden layer. *Supervised learning* is applied, which means that during the training process of a specific *configuration* the weights of the connections are adjusted so as to minimize the error between the actual values of wheel wear or roughness (available in the *experiment files* mentioned above), and the values yielded by the ANN.

The training process has been implemented in Matlab™ 2012b by the Neural Network Toolbox. The non-linear optimization method is Levenberg-Marquardt (Equation (6)) due to its capacity of convergence and efficiency [23]:

(6) $$\Delta x={\left[J^{T}(x)J(x)+\mu I\right]}^{-1}J^{T}(x)e(x)$$

where *J*(*x*) is the Jacobian matrix with the first derivatives of the network errors respect to the network weights and biases, *e* is the network errors vector and *μ* is a parameter that when is large the algorithm become steeps descent and when is small the algorithm becomes Gauss-Newton.

The generalization method is Bayesian Regularization given its generalization capabilities compared to early stopping [24]. In general, the training aim is to reduce the sum squared error (*E_D_*) of the target value and predicted value. Bayesian Regularization adds the sum squared of the network weights (*E_W_*) to the equation [25]:

(7) $$F=\beta E_{D}+\alpha E_{W}$$

where *F* is the objective function and α and β are the objective function parameters. The main problem of the above equation is to find the correct values for *α* and *β*. To solve this problem the Bayesian framework was proposed [24], where the network weights are considered random variables:

(8) $$P\left(\text{w}|D,\alpha ,\beta ,M\right)=\frac{P(D|\alpha ,\beta ,M)P(\text{w}|\alpha ,M)}{P(D|\alpha ,\beta ,M)}$$

where *D* represents de data set, *M* represents the neural network model and **w** is the network weights vector. It is assumed that the noise in the training data set is Gaussian and that the prior distribution for the weights is Gaussian. The optimization of the parameters *α* and *β* require solving the Hessian matrix of *F*(**w**) at the minimum point, which is possible with the Levenberg–Marquardt training algorithm.

Likewise, back-propagation through time BPTT, usually recommended to train recurrent neural networks, is applied [26]. The activation function of the hidden layer is the tangent hyperbolic, characterized by a range of [−1, 1], and the linear activation function is used in the output layer.

For Bayesian regularization it is said that there is no hidden units limit although it is recommended not to have more weights than half the number of training cases (the training cases are 42 in this case) [25]. Considering these indications, one hidden layer with a number of neurons *HU* ranging from 5 to 15 has been tested. As the number of delays *D* is concerned, values from 5 to 15 have been applied. Higher values of *HU* and *D* are not considered *a priori* due to performance purposes from the perspective of both the training time and the resource requirements (memory, processor,…) of the ANN-based sensors during its further operation.

In particular, two training phases are considered in the methodology of the calibration:

1. *Coarse tuning*: during this phase the aim is to obtain the *configurations* (*HU*-*D*) around which the lowest test errors are yielded. Given the *coarse* nature of this phase, the configurations given by the combination of *HU* ∈ {5, 10, 15} and *D* ∈ {5, 10, 15} are trained.
2. *Fine tuning*: the fine tuning of the training process is to be performed in the range given by the best *configurations* inferred in the *coarse* tuning. Considering the *fine* nature of this phase, the values of *HU* and *D* of the new *configurations* to be trained are taken one by one within the specified range.

Each training of the ANN with a particular *configuration* is carried out six times due to the dependence of the error on the initial values of the weights. For the initialization of the weights, the algorithm proposed by Nguyen and Widrow has been applied since it tends to decrease the training time [27].

**Figure 4.**
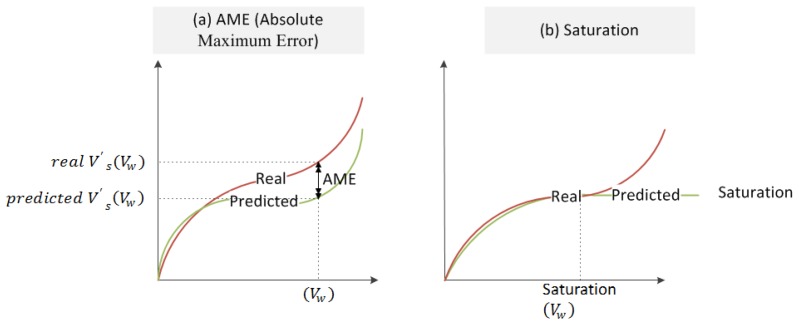
*Ad hoc* indicators (**a**) AME; (**b**) Saturation.

A Training and Test Data Set (TTDS) is generated with a certain combination of the grinding experiments collected in Figure 2 and that are selected for training and testing purposes. A selected subset of grinding experiments is used to train different *configurations* of the ANN (the so-called *training experiments*). The accuracy of a trained *configuration* is computed on another subset of grinding experiments, the so-called *test experiments*, through the following indicators that provide measurements of the deviation between the actual value of the variable (surface finish or wheel wear) and the value provided by the sensor (see Figure 4):

1. The mean square error MSE: it is provided by the Matlab™ training tool and, therefore, its value is given within the normalization range applied during the training process.
2. The absolute maximum error AME: it is computed in units of process (micrometers—μm) as:
  (9) $$\text{AME}=\text{abs}(\max(\text{real}\;{V^{\prime }}_{s}(V_{w})-\text{predicted}\;{V^{\prime }}_{s}(V_{w}))$$
3. Saturation S: the saturation is the phenomenon in which the output stops tracking the target so as the target remains increasing, while the output converges to a mean value. As a consequence, AME tends to infinite.

Since these indicators are computed on the predefined *tests experiments*, *i.e.*, the experiments not used during the training phase, they allow to quantify the generalization capability of the ANN, *i.e.*, the capability of the ANN-based sensors to infer the wheel wear and surface roughness under previously unknown conditions.

**Figure 5.**
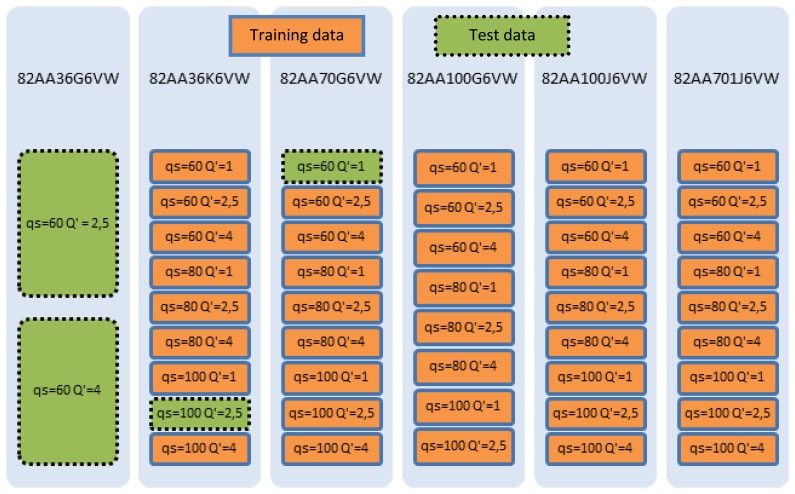
Training and Test Data Set (TTDS).

Actually, the experimental database consists of 46 experiments or sequences of 200 points each. The 200 points of each sequence correspond to subsequent values of *V′_w_* with a sample rate of 10 mm^3^/min yielding a time horizon per experiment equivalent to 2000 mm^3^/min. Thus, power consumption P, wheel wear and roughness are measured every 10 mm^3^/min yielding a total of 200 points each in a time horizon equivalent to 2000 mm^3^/min. For the 46 experiments, 42 experiments of 200 points each are used for training, and four are used for testing. Figure 5 shows this Training and Test Data Set: the training experiments are indicated by orange continuous line rectangles, and the training tests by green dashed line rectangles. It is remarkable that, as explained above, no validation examples are used since Bayesian Regularization is applied. Notice that the *test experiments* have been selected to assess the capability of generalization of the ANN from two points of view. Firstly, the capability of the ANN to estimate the wheel wear/roughness when using a new wheel (in other words, a wheel not used during the training process), but under known cutting conditions (in other words, the cutting conditions have been included during the training process, but with other grinding wheels). In particular, two *test experiments* that correspond to the 82AA36G6VW wheel with *q_s_* 60 and *Q'* 2.5, and *q_s_* 60 and *Q'* 4, respectively, are considered (see Figure 5). Secondly, the capability of the ANN to estimate the wheel wear/roughness when using wheels employed during the training process, but under new cutting conditions. In particular, these test experiments correspond to the 82AA36K6VW wheel with cutting conditions *q_s_* 100 and *Q'* 2.5, and to the wheel 82AA70G6VW with cutting conditions *q_s_* 60 and *Q'* 1 (see Figure 5).

## 3. Discussion of Results of the Calibration Process of the Virtual Sensor

In this section, in order to illustrate the potentiality of the proposed calibration methodology, the analysis and discussion of results is performed on the ANN-based virtual sensor for the estimation of the wheel wear, although the same approach has been applied to the ANN-based virtual sensor for the estimation of the roughness, in this case, average surface roughness (*R_a_*), which is widely used in industry.

**Figure 6.**
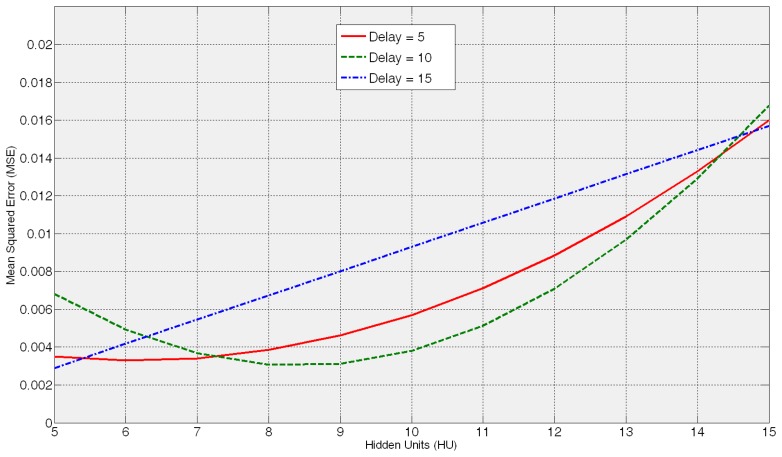
Summary of the results of coarse tuning.

As stated in Section 2, firstly, *coarse* and *fine* tunings are performed with the Training and Test Data Set (TTDS) presented in Figure 5. The *coarse tuning* aims at defining the dimension and the dynamic behaviour of the artificial neural network. The aim is to obtain the *configuration* (*HU*-*D*) around which the lowest test errors are yielded. The lowest test MSE values yielded by the ANNs trained with different combinations of hidden units *HU* and delays *D* are presented in Figure 6. When the delay value is 5 the lowest MSE values are within the range 5–8 hidden units but, nevertheless, for a delay equal to 10, the range shifts to 7–10 hidden units. Finally, although the HU15D5 ANN *configuration* yields a very low MSE value; from 7 *HU* the MSE error increases significantly. Thus, it is concluded that within the ranges 5–10 *HU* and 5–10 *D* is to be found the proper configuration to model the wheel wear behavour. Actually, increasing the number of hidden units and/or delays provides higher test errors that can be associated to the overfitting of those ANNs (Figure 6). Moreover, the additional disadvantage is requiring longer training times.

**Figure 7.**
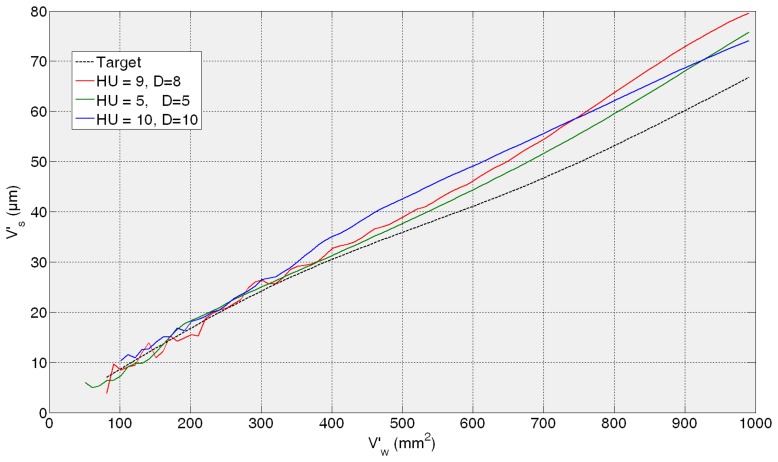
Generalization capability of the three preselected *configurations*. *Test experiment*: 82AA36K6VW *q_s_* = 100; *Q′* = 2.5.

**Figure 8.**
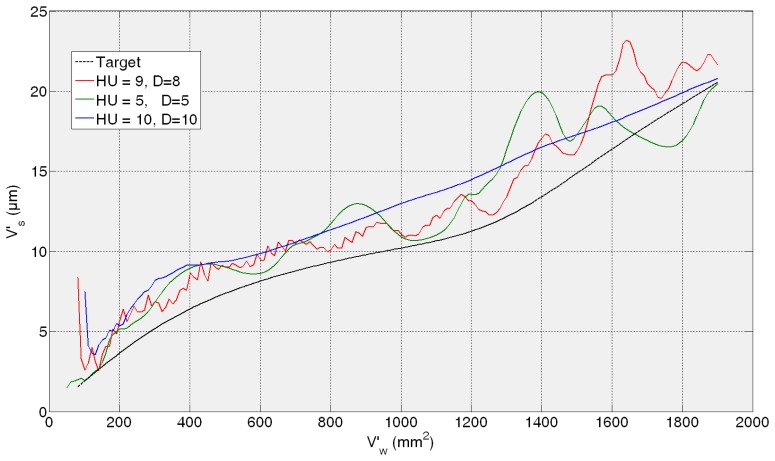
Generalization capability of the three preselected *configurations*. *Test experiment*: 82AA70G6VW *q_s_* = 60; *Q′* = 1.

**Figure 9.**
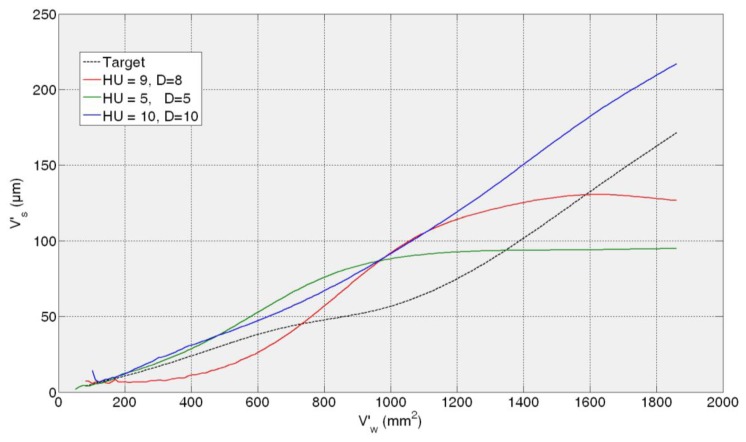
Generalization capability of the three preselected *configurations*. *Test experiment*: 82AA36G6VW *q_s_* = 60; *Q′* = 2.5.

**Figure 10.**
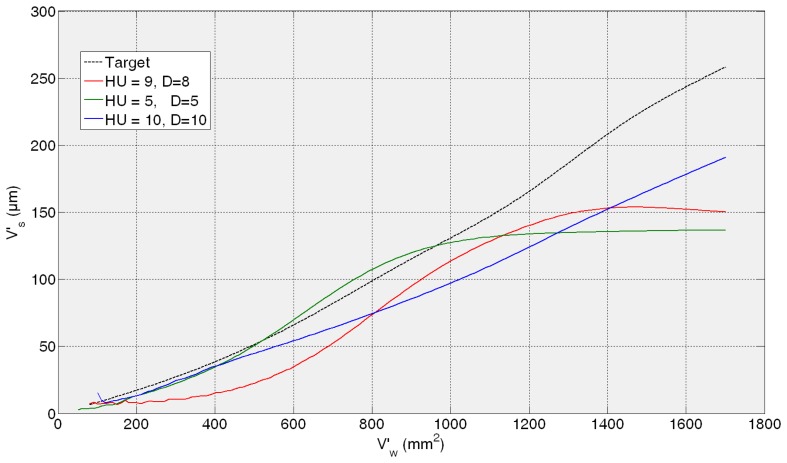
Generalization capability of the three preselected *configurations*. *Test experiment*: 82AA36G6VW *q_s_* = 60; *Q′* = 4.

Then, the *fine tuning* of the training process is performed between 5 and 10 hidden units and 5 and 10 delays. As a summary of the *fine tuning* results, the ANNs that provide the average of the three lowest test error predictions obtained in the fine tuning phase are presented in Table 1. Given MSE values of the same order of magnitude, more than one ANN are preselected so as to analyze the behaviour of the best neural configurations from the grinding process perspective. It can be noticed that all the test MSE values are lower than the best one obtained in the preliminary analysis. The more trainings performed around the proper dimension and timing parameters *HU* and *D*, the more probability to reach lower MSE values. However, the best ANN from the grinding process perspective is not the one with the lowest MSE, *i.e.*, the ANN with a configuration with 10 hidden units and 10 delays (10HU10D) provides the lowest values of AME, with no saturation effect. In order to illustrate it, Figures 7, 8, 9 and 10 show the behaviour of the preselected ANNs when operating with the four *test experiments* of TTDS, as well as the target or real wheel wear values. Notice that there is a gap at the beginning of each signal due to the delay *D* of the net, since the number of previous values specified by *D* is required for the first estimation.

Figure 7 shows the evolution of the estimation of wheel wear for the three preselected ANNs in the first *test experiment* 82AA36K6VW *q_s_* = 100; *Q′* = 2.5. In this case, similar good performance is achieved by the three ANNs; actually, no saturation appears in any case and AME is 13, 9 and 8 μm for 9HU8D, 10HU10D and 5HU5D, respectively, which are considered very good estimations from the grinding process perspective. On the contrary, in the rest of the *test experiments* the differences of performance of the preselected ANNs are more evident.

In the case of the second *test experiment*, Figure 8 shows that the best behaviour is provided by 10HU10D. Although any of the ANNs yield saturated estimations of wheel wear, 9HU8D and 5HU5D provide an oscillatory behaviour of the output with an AME value of 6 μm in both cases, while in the case 10HU10D is 3 μm. Although from the grinding process perspective an error of 6 μm is considered very low in terms of estimation, the oscillatory behaviour of the estimations can indicate a lower generalization capability of the ANNs. Actually, the behaviour of the estimations of the wheel wear is saturated in 9HU8D and 5HU5D for the third and fourth *test experiments* (82AA36G6VW *q_s_* = 60, *Q′* = 2.5, and 82AA36G6VW *q_s_* = 60, *Q′* = 4), while in the case of 10HU10D there is no saturation. Moreover, 10HU10D *configuration* provides the lowest AME value for the third and fourth *test experiments*, which are 49 μm and 67 μm, respectively.

Thus, it can be concluded that the ANN *configuration* 10HU10D provides the most balanced performance over time, which agree with the corresponding values of AME and saturation provided in the results of Table 1. Thus, among the three preselected neural *configurations* shown in Table 1, the ANN *configuration* 10HU10D has been selected, *i.e.*, an ANN based on the *Layer-Recurrent* architecture with one hidden layer consisted of 10 hidden units and with a *memory* equivalent to 10 delays. Other neural configurations, such as HU5D5, provide considerably lower values of AME. However, in some *test experiments* at a given point the output saturates.

From a quantitative point of view, the results show that the initially proposed time horizon (equivalent to 2000 mm^3^/min) is very ambitious. Thus, it is necessary to delimit a time horizon in which the ANN-based sensors behave reliably. Concerning the ANN-based sensor for the estimation of wheel wear, AME ranges in the whole time horizon from 3 to 67 μm, while for a time horizon equivalent to 600 mm^3^/min this range reduces drastically to 3–12 μm, which is very accurate from the grinding process perspective. Also notice that during the time horizon equivalent to 600 mm^3^/min, 390 parts are machined, which is a significant quantity. Nevertheless, from a qualitative point of view the output of the ANN-based sensor tracks the trend of the actual values during the whole time horizon. Concerning the saturation phenomena, notice that the selected network for the estimation of wheel wear (*configuration* 10HU10D: 10 hidden units, 10 delays) does not exhibit saturation phenomena in any of the *test experiments* (*i.e.*, experiments with characteristics not considered during the training process).

Additionally, the results show that better performance is achieved when the virtual sensor estimates the behaviour of a wheel employed during the training process, but under new cutting conditions (see Figures 7 and 8). Actually, the performance of the virtual sensor decreases when predicting the behaviour of a new wheel (a wheel not used during the training process), but under known cutting conditions (see Figures 9 and 10). This means that the characteristics of the wheel have a higher influence on the wear behaviour than the cutting parameters.

A final remark can be done from the point of view of industrial application. For many users and also for many grinding wheel manufacturers, a single numerical indicator is used to analyse the performance of wheel from the point of view of wear over time. This is known as the *grinding ratio G*, which is defined in Equation (10):

(10) $$G=\frac{\Delta V_{w}}{\Delta V_{s}}$$

This parameter can be additionally used for assessing the quality of the tuning process of the new sensor. When looking at wear patterns along time, an initial short stage during which wear rate is very fast can be observed. Afterwards, it is accepted [22] that linear behaviour prevails, and it is during this stage that *G* is measured. Therefore, grinding ratio values obtained from actual wear measurement during experimental tests and those produced by the virtual sensor have been calculated and compared. Results have been collected in Table 2.

Table 2 shows the grinding ratio (*G*) results of the four *test experiments*. The results are consistent with the wheel wear results and show that the best ones are achieved with the wheels used during training but under new cutting conditions. For the first and second *test experiments* the errors are 2 and 10, respectively. These errors are very satisfactory from an expert on grinding point of view showing the potential of the presented sensor. In the case of a new wheel (third and fourth *test experiments*), the error is 2 for both *test experiments*. The absolute errors are considered low for the grinding process perspective in all the cases, but the relative ones are a bit higher for the third and fourth *test experiments*. Therefore, as mentioned above, it can be clearly seen that the characteristic of the wheels have higher influence on the wheel wear prediction and, consequently, on the generalization capabilities of the ANN-based sensor.

## 4. Industrial Validation of the ANN-Based Virtual Sensor: Application Examples

In this section, analysis of the performance of the new sensor during actual industrial application is presented. Since, as said before, the methodology for wear and roughness virtual sensing is identical, in this Section results of the ANN-based virtual sensor for on-line monitoring of roughness (*R_a_*) are commented on. Therefore, the neural network has been trained using the data base presented in Section 2.3, yielding a neural network *configuration* 8HU8D, which is very close to the one obtained for the ANN-based virtual sensor for measuring wheel wear (Figure 6). Thus, it is well demonstrated that a neural *configuration* with an order of magnitude near to 10 hidden units and 10 delays allows to properly model the relationships in the scope of the grinding process, as shown in Equations (1) and (2).

Table 3 shows the summary of the results obtained with the four *test experiments*. It can be noticed that AME ranges from 0.26 to 0.47 μm, which are acceptable errors from the perspective of the grinding process itself.

**Figure 11.**
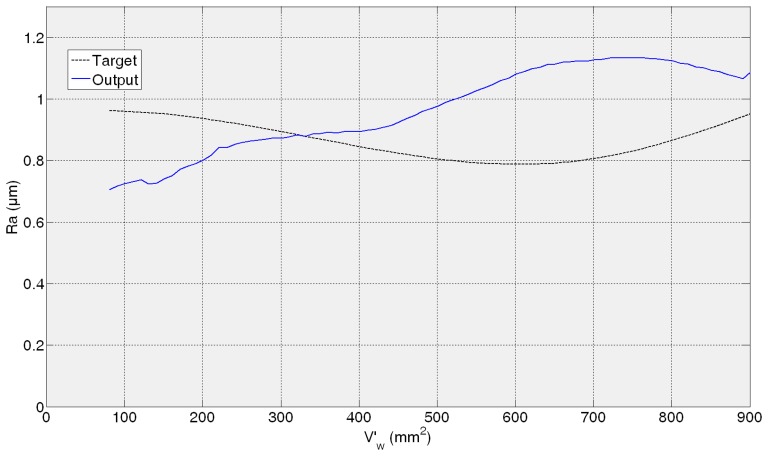
*R_a_* measurement for 82AA36K6VW wheel, *q_s_* = 100 and *Q′* = 2.5 grinding process variables.

**Figure 12.**
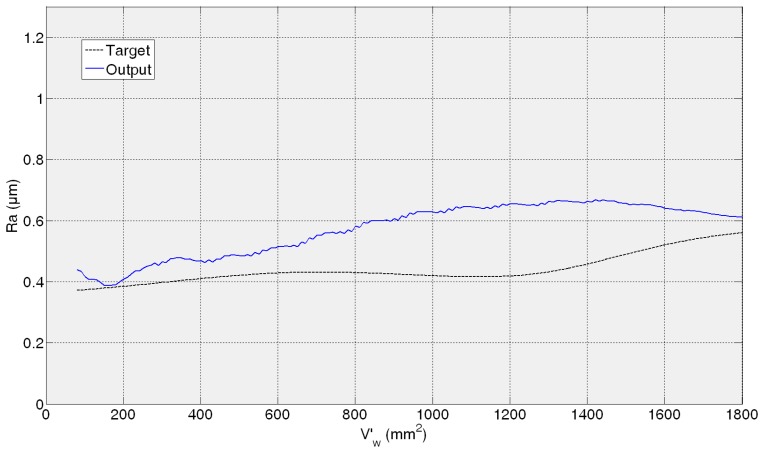
*R_a_* measurement for 82AA70G6VW wheel, *q_s_* = 60 and *Q′* = 1 grinding process variables.

**Figure 13.**
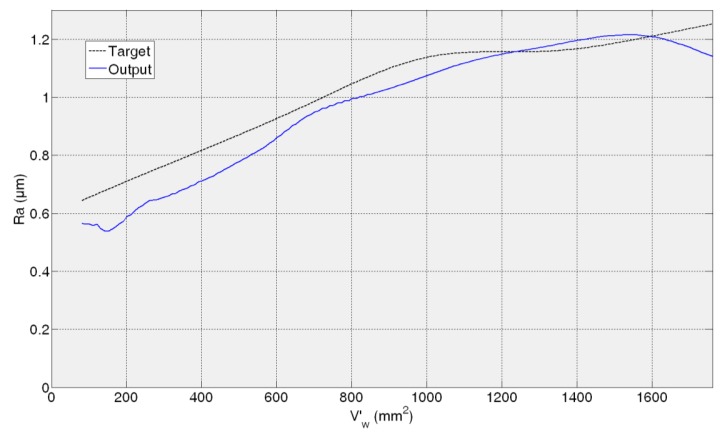
*R_a_* measurement for 82AA36G6VW wheel, *q_s_* = 60 and *Q′* = 2.5 grinding process variables.

**Figure 14.**
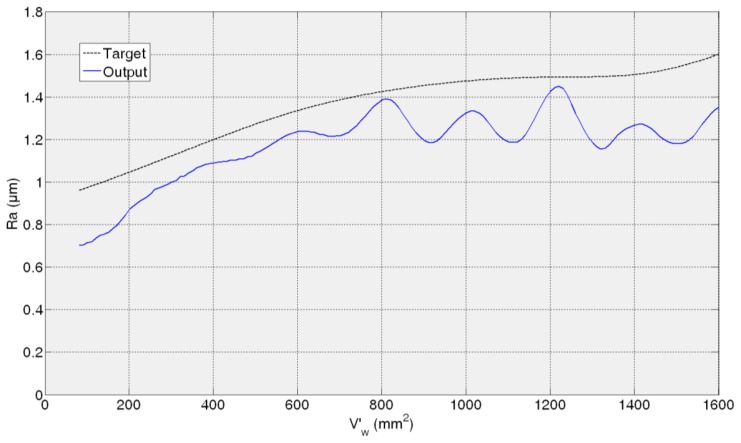
*R_a_* measurement for 82AA36G6VW wheel, *q_s_* = 60 and *Q′* = 4 grinding process variables.

Figures 11, 12, 13 and 14 show both the real values (available in the *experiment files*) and the measured values by the ANN-based virtual sensor for roughness in the four *training tests*, respectively. The noticeable oscillation of the output of the ANN-based sensor can be attributed to the scale of the roughness values, with variations from point to point of less than 0.1 μm. This is why in the surface finish virtual sensor, an additional function has been implemented, which is a sliding average every 10 sensed data.

Figure 11 shows the evolution of *R**_a_*** for the 82AA36K6VW grinding wheel when process variables are *q_s_* = 100 and *Q′* = 2.5 (first *test experiment*). It can be noticed that at the beginning, sensed *R**_a_*** is lower than the experimentally measured value. When more material is removed, the *R**_a_*** value yielded by the virtual sensor increases and is higher than the real one. Finally, at the end both values, sensed and real, are similar. In Figure 12 the behavior is similar; *R**_a_*** as measured by the virtual sensor is higher than the experimental one. In Figure 12 the evolution for 82AA70G6VW wheel and *q_s_* = 60 and *Q′* = 1, second *training test*, is shown. Both wheels were employed during training, but under new grinding conditions. No saturation appears in both cases and AME is 0.32 μm and 0.26 μm for 82AA36K6VW *q_s_* = 100 and *Q′* = 2.5; and 82AA70G6VW *q_s_* = 60 and *Q′* = 1, respectively.

In the case of the third test experiment (82AA36G6VW wheel, *q_s_* = 60 and *Q′* = 2.5) the signal provided by the sensor follows pretty well the real one nearly until the end of the experiment (*V*'*_w_* = 1600 mm^2^) (Figure 13). Actually, AME is low, 0.23 μm. Figure 14 shows the evolution of real and sensed *R_a_* for the fourth test experiment (82AA36G6VW wheel, *q_s_* = 60 and *Q′* = 4). At the beginning the signal from the virtual sensor acceptably follows the real one. However, around *V'_w_* equal to 700 mm^2^ the signal from the sensor starts oscillating. As explained above, it can be attributed to the scale of the roughness values (even though the sliding average has been applied) and to a lower generalization capability of the ANN. In this case, AME is 0.47 μm. Both cases, third and fourth test experiment, are under known cutting conditions but with a new wheel which had not been used during the training process of the ANN.

Similar to the ANN-based sensor for the estimation of wheel wear, in the case of the ANN-based sensor for the estimation of roughness, AME ranges in the whole range from 0.23 to 0.47 μm, while for a time horizon equivalent to 600 mm^3^/min this range reduces to 0.06–0.3 μm. These are considered satisfactory estimations from the grinding process perspective. Moreover, it must be taken into account that, in the experimental measurement of surface roughness using the surface measuring device, a certain dispersion is always present (in particular, the values of AME given above are computed with respect to the mean value of four measurements, which is a common practice in grinding).

In contrast to wheel wear, in *R**_a_*** monitoring the performance of the soft sensor does not decrease for all the cases with a new wheel. Actually, the best performance is achieved for the third test experiment. This shows that for surface roughness estimation the wheel does not have such an influence on the surface finish behaviour unlike for wheel wear estimation. Besides, one could think that grinding energy has a higher influence on surface roughness modelling than on wheel wear modelling.

Finally, compared to the wheel wear case, in which the signal always starts at zero (*i.e.*, at the beginning there is no wear), the sensor is capable to precisely estimate the initial roughness of each *test experiment*. Therefore, the proposed methodology can model two signals (wheel wear and surface roughness) characterized by completely different behaviours. Concerning the initial error in the prediction of the surface roughness, in the worst case (fourth *test experiment*), the error is 0.26 μm, which is a low prediction error from the grinding process perspective.

## 5. Conclusions

In this paper a new virtual sensor for wheel wear and workpiece surface roughness measurement during the grinding process has been presented. The proposal is based on a virtual sensor that makes use of the modelling ability of Artificial Neural Networks. As a result, the sensor success in on-line monitoring of both variables shows the capability of the ANN to estimate the wheel wear/roughness when using a new wheel, but under known cutting conditions, and the capability of the ANN to estimate the wheel wear/roughness when using wheels employed during the training process, but under new cutting conditions. The ANN-based virtual sensor supports on a very simple and low-cost power meter device (that can be easily implemented in any kind of grinding machine) used as on-line input signal for the virtual sensor, avoiding thus the need for expensive force measuring devices (such as the dynamometric table) or the need for machine mechanical modification.

On these foundations, two sensors have been implemented, one for wheel wear monitoring and other for surface roughness. From the work carried out, the following conclusions can be drawn:

- Obtaining on-line values of surface roughness and wheel wear during the grinding process is a very difficult task. A virtual, ANN-based sensor which uses power meter values from wheel spindle has been designed, calibrated and its performance has been evaluated in industrial tests.
- The selected ANN architecture, *Layer*-*Recurrent* Neural Network, has shown the potential for estimating wheel wear and surface roughness without measuring initial real values up to 2000 mm^2^ of specific volume of part material removed. The highest AME error for wheel wear is 67 μm and 0.47 μm for surface roughness.
- A calibration methodology has been proposed and tested for the new soft sensor. Calibration process involves establishing the optimum values of delays and hidden units for the ANN. This is carried out in a two-stage process, which involves *coarse* and *fine* tuning of the sensor.
- It is not possible to select the best ANN *configuration* only by comparing MSE values. Actually, the HU5D5 ANN *configuration* has achieved the best MSE value but it does not provide the best behavior for estimating the grinding process variables. Thus, two new *ad*-*hoc* indicators are proposed: the AME value and saturation. Those indicators help to select the best *configuration* in a *fine* tuning calibration stage.
- The lowest errors (and therefore, the best performance of the sensor) are achieved with a neural *configuration* centered in 10 hidden units and 10 delays. In other words, this is the network *configuration* that optimally represents the grinding process. This result is confirmed for modelling both wheel wear and surface roughness, confirming thus the initial hypothesis accepted by literature and expressed in Equations (1) and (2).
- In wheel wear monitoring the characteristics of the wheel have a higher influence on the wear behaviour than the cutting parameters. In fact, the highest AME values for wheels used and not used during the training process are 9 μm and 67 μm, respectively.
- For surface finish estimation, the wheel does not have such an influence on the surface finish behaviour. Actually, similar AME values have been obtained for both used and new wheels: in particular, an AME 0.26 μm for a used wheel, and 0.23 μm for a new wheel. Even more, the lowest AME has been yielded by a *test experiment* performed on a new wheel. One could think that the reason for this effect is that the grinding energy has a higher influence on surface roughness modelling than on wheel wear modelling.

## Figures and Tables

**Table 1.** Summary of the fine tuning results.

**Hidden Units (HU)**	**Delays (D)**	**AME (μm)**	**Saturation**
9	8	43	Yes
10	10	32	No
5	5	53	Yes

**Table 2.** Summary of grinding ratio (*G*) results of the test experiments.

	**Experimental**	**Sensed**
82AA36K6VW *q_s_* = 100; *Q′* = 2.5	16	14
82AA70G6VW *q_s_* = 60; *Q′* = 1	100	110
82AA36G6VW *q_s_* = 60; *Q′* = 2.5	10	8
82AA36G6VW *q_s_* = 60; *Q′* = 4	6	8

**Table 3.** Summary of results of the test experiments.

	**AME (μm)**	**Saturation**
82AA36K6VW *q_s_* = 100; *Q′* = 2.5	0.32	No
82AA70G6VW *q_s_* = 60; *Q′* = 1	0.26	No
82AA36G6VW *q_s_* = 60; *Q′* = 2.5	0.23	No
82AA36G6VW *q_s_* = 60; *Q′* = 4	0.47	No
